# Odor lateralization test is insensitive to small degrees of intranasal trigeminal activation

**DOI:** 10.1007/s00405-024-09016-x

**Published:** 2024-10-08

**Authors:** Yiling Mai, Benjamin Brieke, Thomas Hummel

**Affiliations:** Smell and Taste Clinic, Department of Otorhinolaryngology, Faculty of Medicine Carl Gustav Carus, Technische Universität Dresden, Fetscherstraße 74, 01307 Dresden, Germany

**Keywords:** Odor lateralization, Trigeminal, Olfactory, Odor intensity, Bimodal odor

## Abstract

**Introduction:**

Odors with prominent trigeminal compounds are more easily localized than purely olfactory ones. However, it is still unclear whether adding a small amount of a trigeminal compound to an olfactory odor significantly improves lateralization performance.

**Methods:**

We included 81 healthy adults aged 25.4 ± 4.8 years to complete odor lateralization tasks using 12 odors: two “olfactory”, two “trigeminal” odors, and eight odor mixtures at two low concentrations of “trigeminal” odors (4%, 8%). This task utilized a “Squeezer” delivering odor or air to either nostril, and participants indicated which nostril received the odor. Evaluations also included olfactory function, odor intensity ratings, and individual olfactory importance.

**Results:**

Degrees of trigeminal compounds significantly affected lateralization performance (F = 82.32, *p* < 0.001), with 100% irritants showing higher performance than 0%, 4%, and 8% irritants (p’s < 0.001), while no significant differences were found between odors with 0%, 4%, and 8% irritants (p’s > 0.05). Chi-square tests confirmed higher percentages of above-chance lateralization with 100% irritants than with 0%, 4%, and 8% irritants (χ2 = 30.89 to 47.33, p’s < 0.001).

**Conclusions:**

Adding a small amount of a trigeminal compound to a selective olfactory odor does not significantly improve lateralization performance. Trigeminal lateralization likely follows an “accumulative” pattern rather than an “all or none” rule. With only 20 trials, the task may lack sensitivity to detect low levels of trigeminal irritation in selective olfactory odors, though it does not rule out trigeminal activation. The odor lateralization task can screen for odors with prominent trigeminal compounds by comparing group-level performance with that of purely olfactory odors. Future studies should use more ideal stimuli (e.g., PEA for olfactory, CO2 for trigeminal) to test the replicability of the results.

## Introduction

The human nasal cavities contain two closely connected sensory pathways: the olfactory and trigeminal systems, which together contribute to odor perception [1]. The olfactory system is responsible for detecting and identifying odor molecules, while the trigeminal system mediates somatosensations such as irritation, temperature, and airflow. As a result, the trigeminal system can trigger protective reflexes, such as sneezing, when noxious substances are detected [2, 3].

A common method for studying the trigeminal system is the lateralization paradigm, in which an odorant is delivered to one nostril and an odorless stimulus to the other [4, 5]. Participants are then asked to indicate which nostril received the odor. This approach relies on the fact that odor lateralization requires trigeminal activation, as humans generally cannot lateralize odors based on olfactory input alone [6, 7]. For example, Kobal et al., [4] used an olfactometer to deliver olfactory or trigeminal stimuli to the participants’ left or right nostril. They found that participants localized “pure” odorants like hydrogen sulfide or vanillin at chance level, while trigeminal stimuli like carbon dioxide or menthol yielded over 96% correct lateralization.

However, enhancing the ability to localize “pure” odorants is not entirely impossible. This can be achieved through training or by mixing “pure” odorants with “trigeminal” odors (refer to odors that have trigeminal activity) [8, 9]. In a previous study, Tremblay et al. compared lateralization performance between “pure” odorants, “trigeminal” odors, and mixtures of the two at ratios of 1:1 or 2: 1. They found that lateralization of mixtures was significantly better than that of “pure” odorants [9]. This raises a subsequent question of how sensitive lateralization tasks are to trigeminal activation. Can even a small addition of a “trigeminal” odor to an “olfactory” odor significantly enhance lateralization performance? In other words, does odor lateralization follow an “all or none” rule or an “accumulative” pattern? If the “all or none” rule holds true, lateralization performance would significantly improve even with the addition of a very low level of trigeminal irritation to a selectively “olfactory” odor. On the other hand, if the “accumulative” pattern is true, odor lateralization performance would only significantly improve for an odor with a trigeminal component prominent enough to elicit irritation.

We thus aimed to investigate whether odor lateralization performance significantly improved when “olfactory” odors were mixed with varying small degrees of irritating (trigeminal) compounds. We also explored factors correlated to lateralization performance. By answering the “all or none” question, we aimed to provide insights into the interaction between olfactory and trigeminal sensations and evidence of the odor lateralization task’s sensitivity in discriminating or selecting odors with different degrees of trigeminal activation.

## Methods

### Participants

We recruited healthy adult volunteers who reported a normal sense of smell, and confirmed through the Sniffin’ Sticks Identification test [10, 11]. Exclusion criteria were: (a) pregnancy or lactation, (b) significant health impairments (e.g., Parkinson’s disease), (c) acute or chronic inflammation of the nose or sinuses, and (d) smoking more than 5 cigarettes per week. Informed consent was obtained from all participants. The study was approved by the Ethics Committee at the Medical Faculty of TU Dresden (EK278082019) and was conducted in accordance with the Declaration of Helsinki.

### Procedure

We scheduled the tasks over three appointments within a two-week period to ensure a manageable workload for participants. In the first appointment, participants underwent an assessment, including olfactory function tests, questionnaires on demographic information, and lateralization tests with five out of the twelve odors in a randomized sequence. Participants also rated the intensity of these five odors. In the second appointment, participants filled out a questionnaire regarding the individual importance of olfaction, another five lateralization tests and odors intensity ratings. The sequence of odors assessed with the lateralization tests was also randomized. In the third appointment, participants completed lateralization tests using the remaining two olfactory odors in a randomized sequence and rated their intensity.

### Measurements

***Odor lateralization test.*** This test employs a mechanically operated stimulation device commonly known as a “squeezer” [4, 12, 13]. Two compressible polypropylene bottles, each with a capacity of 250 ml, were placed within the squeezer. One bottle was filled with an odorant, while the other remained empty. A conical outlet at the necks of the bottles connected to a piece of silicone tubing with an inner diameter of 4 mm, which served as a nose piece. During the test, an odor stimulus was presented to one nostril, while the other nostril received air from the second bottle. Each squeeze resulted in a 15 ml volume per bottle. Participants were blindfolded and instructed to raise either their left or right hand to indicate the side of stimulation after each squeeze. A total of 20 stimuli (10 for each nostril) were presented in a randomized order, with an interval of 30s between trials. The score was calculated as the sum of correct responses.

***Odors used in the lateralization test***. In total, 12 odors were tested: 2 “olfactory” (O1, O2), 2 “trigeminal” (T1, T2), and 8 mixtures, where O1 and O2 were separately combined with small percentages (4% and 8%) of the T1 and T2. A pilot test with simple questions of irritability, intensity, and pleasantness confirmed that the T1 and T2 were distinctly perceived as irritating, while the O1 and O2 were perceived without irritation. The concentrations of T1 and T2 (4% and 8%) were determined as small degrees of irritants because they did not noticeably increase perceived irritancy when mixed with olfactory odors. Further details are provided in Table 1.

**Table 1:** Odors used in the lateralization test

Odorant name	Odor type	Odor description
O1	“Olfactory” odorant	Current market product “Glade Tranquil Lavender and Aloe”, a blend of lavender with aloe (component: https://www.whatsinsidescjohnson.com/us/en/brands/glade/Glade--PlugIns--Scented-Oil-Refills---Tranquil-Lavender---Aloe)
O2	“Olfactory” odorant	Current market product “Glade Vanilla Passion Fruit”, a blend of vanilla with passion fruit (https://www.whatsinsidescjohnson.com/us/en/brands/glade/PlugIns-Scented-Oil-Refills-Vanilla-Passionfruit)
T1	“Trigeminal” odorant	Commercial fragrance “Freshness Brick”, a blend of 49.75% l-menthol, 49.75% methanediol, and 0.5% vanillyl ethyl ester to 30% in DPMA (dipropylene glycol methyl ester acetate)
T2	“Trigeminal” odorant	Commercial fragrance “TechNeat”, a blend of 47.5% l-menthol, 47.5% dimene (a mixture of substances mainly containing 3,7-dimethylocta-1,6-diene), and 5.0% lime oxide (a mixture of: 1,6-octadien-3-ol, 3,7-dimethyl-, acid-isomerized) to 30% in DPMA
O1T1_4	Olfactory-trigeminal mixture	96% O1 mixed with 4% T1
O1T1_8	Olfactory-trigeminal mixture	92% O1 mixed with 8% T1
O1T2_4	Olfactory-trigeminal mixture	96% O1 mixed with 4% T2
O1T2_8	Olfactory-trigeminal mixture	92% O1 mixed with 8% T2
O2T1_4	Olfactory-trigeminal mixture	96% O2 mixed with 4% T1
O2T1_8	Olfactory-trigeminal mixture	92% O2 mixed with 8% T1
O2T2_4	Olfactory-trigeminal mixture	96%O2 mixed with 4% T2
O2T2_8	Olfactory-trigeminal mixture	92% O2 mixed with 8% T2

Note. The odors were provided by Takasago International Corporation (Paris, France). Commercial products were used in this study because it was part of a larger project aimed at examining daily life fragrances and their impact on various behavioral outcomes, such as memory. Based on simple pilot questions of irritancy, T1 and T2 were distinctly perceived as irritating, while the O1 and O2 were perceived without irritation. The concentrations of T1 and T2 (4% and 8%) were determined because they did not noticeably increase perceived irritancy when mixed with “olfactory” odors

***Odor intensity.*** Participants rated the intensity of all tested odors using a scale from 0 (no perception) to 10 (extremely intense).

***Olfactory function.*** We used the Sniffin’ Sticks odor identification (OI) to assess olfactory function (Burghart, Holm, Germany) [10]. The maximum score for each test is 16. Scores ≥ 11 is considered a normosmia [11].

***The importance of olfaction.*** The Importance of Olfaction Questionnaire (IOQ) was utilized to assess the significance of olfaction in daily life [14]. This 20-item questionnaire uses Likert scale ratings to measure various aspects of how individuals perceive and use their sense of smell. Higher scores indicate that the individual attributes greater importance to their sense of smell.

### Data analysis

Data were analyzed using SPSS 29.0 software (IBM Corp., Armonk, NY, USA). Descriptive analyses described demographic information and study measurements. A generalized linear mixed model (GLMM) examined the effects of degree of irritant (0%, 4%, 8% and 100%), odorant type (O1 and O2), irritant type (T1 and T2), and their two-way and three-way interactions on odor lateralization performance. Post-hoc comparisons were conducted with sequential Bonferroni correction. To handle missing data from dropouts, GLMM was conducted with Satterthwaite method to correct the degrees of freedom [15–17]. Furthermore, since achieving an above-chance cutoff (≥ 15 points) is a typical indicator in lateralization tasks, we used Chi-square tests to compare the percentage of participants reaching this threshold across different odor conditions. A binomial test confirmed that achieving ≥ 15 correct responses out of 20 trials was significantly higher than chance (test level = 0.50, p = 0.041) [7]. Pearson correlation analysis was used to examine the correlations between the lateralization test scores and odor intensity ratings, Sinffin’ Stick identification score, and the significance of smell. All statistical tests were two-sided, with α = 0.05 considered statistically significant.

## Results

We initially recruited 81 participants (mean age 25.4 ± 4.8 years, 51 women), all with Sniffin’ Sticks identification scores above the normosmic cutoff (≥ 11), with a mean score of 14.43 ± 1.18. Due to dropouts, participant numbers varied across the four appointments: 80 attended the second, 53 completed the third. Thus, at least 53 participants completed the lateralization tasks for all tested odors. There were no significant demographic differences between dropout (*n* = 28) and non-dropout (*n* = 53) participants: age (25.79 ± 4.91 vs. 25.23 ± 4.75, t = 0.50, *p* = 0.62), gender distribution (21/7 vs. 30/23 [female/male], χ²=2.66, *p* = 0.10), BMI (22.29 ± 2.95 vs. 22.08 ± 3.28, t = 0.28, *p* = 0.78), and odor identification scores (8.22 ± 1.85 vs. 7.87 ± 2.02, t = 0.24, *p* = 0.83). Further, to handle missing data, GLMM with corrections for degrees of freedom using the Satterthwaite method was used, thus all samples were accounted for the analysis [15]. Descriptive results are presented in Table 2.

**Table 2:** Descriptive results

	Mean/Counts	SD/Percent
**Age (** ***n*** ** = 81)**	25.42	4.79
**Gender (** ***n*** ** = 81)**		
Women	51	63.00%
Men	30	37.00%
**BMI (** ***n*** ** = 81)**	22.15	3.15
**IOQ (** ***n*** ** = 81)**	35.7	7.19
**OI (** ***n*** ** = 81)**	14.43	1.18
**Lateralization test (0–20)**		
O1 (*n* = 53)	13.06	4.08
O2 (*n* = 53)	12.11	3.98
T1 (*n* = 80)	16.26	3.51
T2 (*n* = 81)	16.68	3.27
O1T1_4 (80)	12.83	3.63
O1T1_8 (*n* = 81)	13.72	3.75
O1T2_4 (*n* = 80)	13.28	3.84
O1T2_8 (*n* = 80)	12.95	4.11
O2T1_4 (*n* = 80)	13.2	3.8
O2T1_8 (*n* = 80)	11.99	3.72
O2T2_4 (*n* = 81)	12.98	3.77
O2T2_8 (*n* = 81)	12.53	4.03
**Intensity (0–10)**		
O1 (*n* = 53)	6.3	2.04
O2 (*n* = 53)	5.69	2.27
T1 (*n* = 80)	8.27	1.74
T2 (*n* = 81)	8.18	1.73
O1T1_4 (*n* = 80)	6.04	2.07
O1T1_8 (*n* = 81)	6.33	2.07
O1T2_4 (*n* = 80)	6.13	2.09
O1T2_8 (*n* = 80)	5.85	2.11
O2T1_4 (*n* = 80)	5.96	2.28
O2T1_8 (*n* = 80)	5.79	2.13
O2T2_4 (*n* = 81)	5.80	2.15
O2T2_8 (*n* = 81)	5.68	2.13

Note. BMI = Body mass index; SD = Standard Deviation; IOQ = Importance of Olfaction Questionnaire. O1 = Olfactory odor 1, O2 = Olfactory odor 2; T1 = Trigeminal odor 1, T2 = Trigeminal odor 2; O1T1_4 = 96%O1 + 4%T1; O1T1_8 = 92%O1 + 8%T1; O1T2_4 = 96%O1 + 4%T2; O1T2_8 = 92%O1 + 8%T2; O2T2_4 = 96%O2 + 4%T2; O2T2_8 = 92%O2 + 8%T2

### Lateralization performances across tested odors

**Fig. 1:**
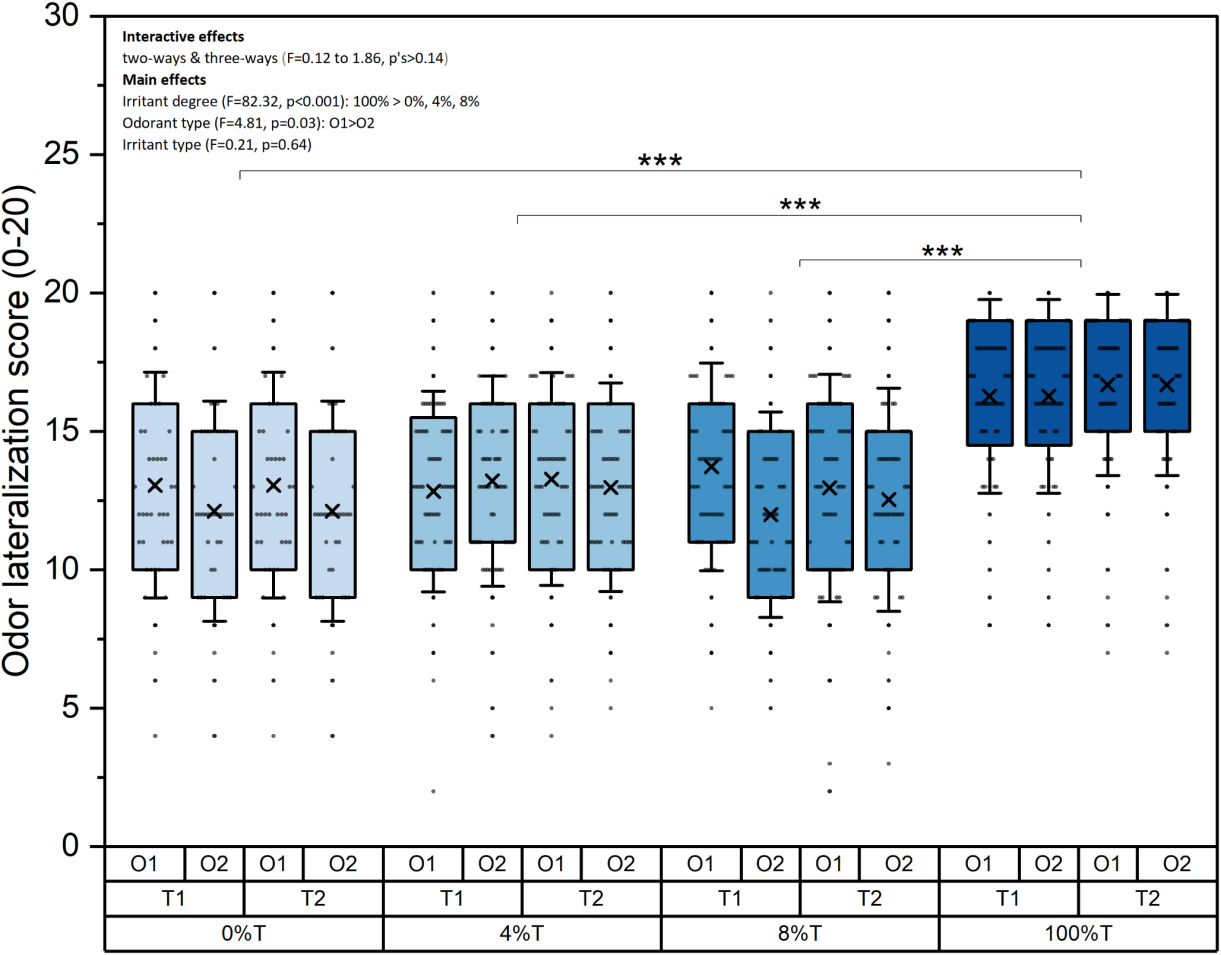
The lateralization scores among odors with different types and degrees of trigeminal compounds mixed with different olfactory odors. Note. The box plots display the lateralization scores of using different combinations of odors. The boxes indicate the interquartile range, with a cross representing the mean value. Standard deviation is presented with the upper and lower whiskers. Data for each subject are shown as dots. O1=“olfactory” odor 1, O2=“olfactory” odor 2; T1=“trigeminal” odor 1, T2=“trigeminal” odor 2; 0%, 4%, 8%, and 100% represent the percentage of trigeminal odors added to the olfactory odors. *** *p* < 0.001

As shown in Fig. 1, GLMM revealed significant effects for “irritant degree (0% vs. 4% vs. 8% vs. 100%)” (F = 82.32, *p* < 0.001) and “odorant type (O1 vs. O2)” (F = 4.81, *p* = 0.03), but no significant effects for “irritant type” or any two- or three-way interactions (F = 0.12 to 1.86, p’s > 0.05). Post hoc tests for “irritant degree” indicated that lateralization performance with 100% trigeminal irritants (M ± SE, 16.47 ± 0.19) significantly outperformed 0% (12.59 ± 0.28), 4% (13.07 ± 0.21), and 8% (12.80 ± 0.22) trigeminal irritants (t = 11.60 to 12.75, p’s < 0.001). However, no significant differences of lateralization performance were observed between 0%, 4%, and 8% trigeminal irritants (t = 0.60 to 1.39, p’s > 0.05). Additionally, post hoc tests for “odorant type” revealed that lateralization performance with odors containing O1 (13.98 ± 0.16) was significantly better than odors containing O2 (13.48 ± 0.16; t = 2.19, *p* = 0.03).

**Fig. 2:**
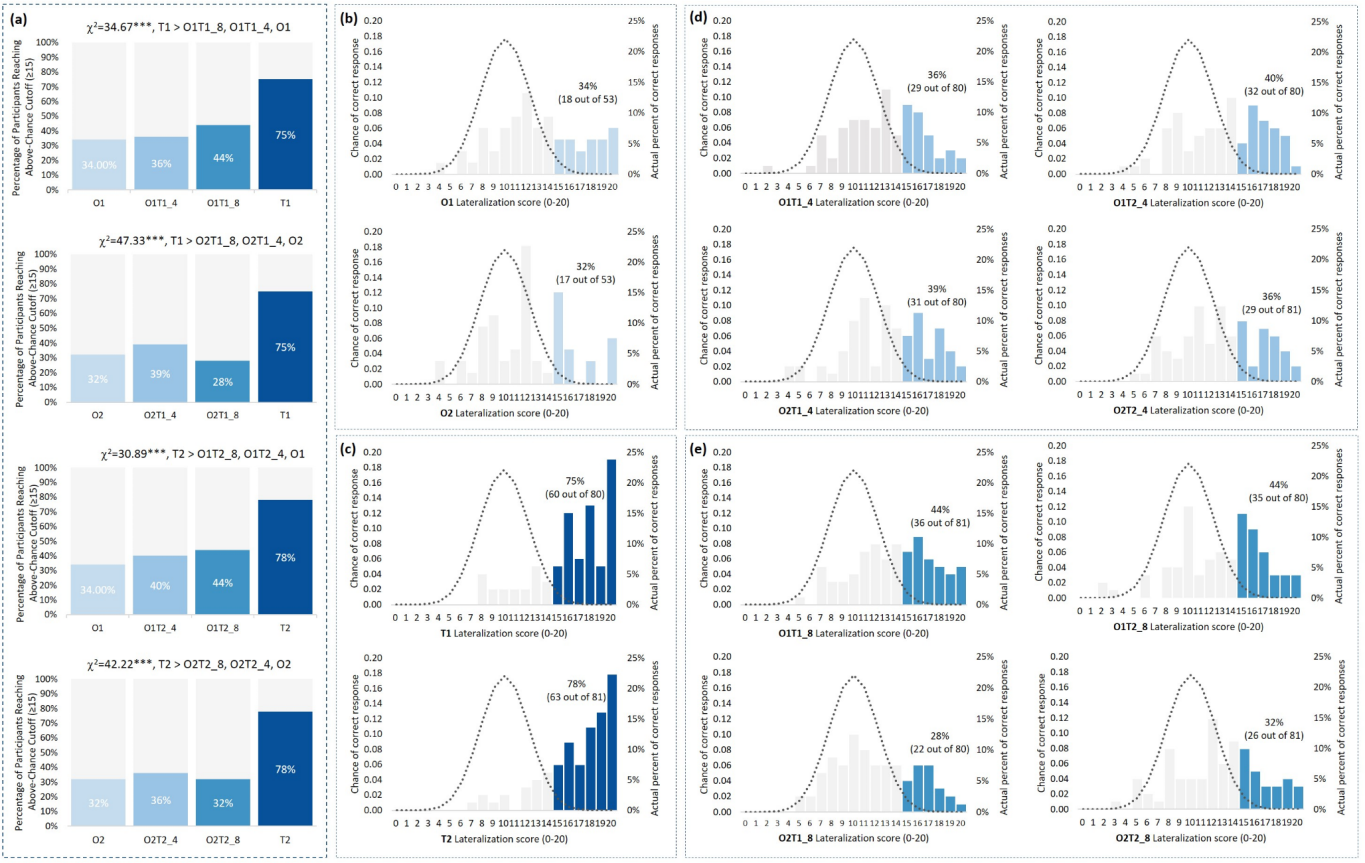
Percentage of participants reaching above-chance threshold (≥ 15) across different types of odors. Note. Figure (**a**): Percentages of participants achieving above-chance lateralization performance (≥ 15 correct responses) across different irritant concentrations (0%, 4%, 8%, and 100%) with various trigeminal-olfactory odor combinations. Significant results from Chi-square tests with post-hoc analysis are indicated above the bars. ***: *p* < 0.001. Figures (**b**-**e**): Distribution of lateralization scores for each odor condition: (**b**) “Olfactory” odors; (**c**) “Trigeminal” odors; (**d**) Mixed odors with 4% irritants; (**e**) Mixed odors with 8% irritants. The left y-axis represents the theoretical probabilities of correctly responding to 0–20 items (binomial distribution), and the right y-axis shows the actual percentage of correct responses. The x-axis denotes the number of items. The dashed line corresponds to the binomial curve, referencing the left y-axis. The bar chart represents the actual percentages of correct responses, referencing the right y-axis. Darker bars indicate the percentages of participants scoring ≥ 15 (above-chance cutoff)

In addition, the percentage of participants reaching the above-chance cutoff (≥ 15 points) was analyzed across different levels of trigeminal irritants. As shown in Figs. 2 and 75% (60 out of 80) and 78% (63 out of 81) of participants met this threshold when exposed to 100% irritants (T1 and T2). In contrast, only 34% (18 out of 53) and 32% (17 out of 53) did so with 0% irritants (O1 and O2), and similarly, 28–44% (22 to 35 out of 80, 26 to 36 out of 81) reached this threshold with 4% or 8% trigeminal irritants. Chi-square tests confirmed that the percentage of participants reaching the cutoff varied significantly across different degrees of irritants (χ²=30.89 to 47.33, p’s < 0.001), with a higher percentage of participants reaching this threshold in the 100% irritants condition compared to the 0%, 4%, and 8% irritants conditions (p’s < 0.05).

Although a higher percentage of participants were able to lateralize T1 or T2 with scores ≥ 15, a small proportion (31 out of 81) could not, indicating some variability within the sample. Therefore, it is important to examine whether the observed effect of “irritant degree” in the overall sample also applies to this subgroup. GLMM revealed a significant effect of “irritant degree” on lateralization performance (F = 7.72, *p* < 0.01), with 100% irritants (13.45 ± 0.29) significantly outperforming both 8% (11.45 ± 0.34; t = 4.47, *p* < 0.01) and 4% irritants (11.90 ± 0.33; t = 3.53, *p* < 0.01), and closely approaching but not reaching significance compared to 0% irritants (12.23 ± 0.39; t = 2.51, *p* = 0.051). Similarly, a small proportion of participants (24 out of 53) were able to lateralize O1 and O2 better than above-chance cutoff. GLMM again showed a significant effect of “irritant degree” (F = 16.91, *p* < 0.01), with performance in the 100% irritants condition (16.73 ± 0.31) significantly better than with 8% (13.77 ± 0.37), 4% (14.02 ± 0.35), and 0% (15.33 ± 0.31) irritants (t = 2.85 to 6.12, p’s < 0.05).

### Correlation results

We observed significant correlations between performance with mixed odors and their corresponding intensity ratings: (1) O1T1_8 lateralization score positively correlated with O1T1_8 intensity (*n* = 81, *r* = 0.30, *p* < 0.01); (2) O1T2_4 lateralization score positively correlated with O1T2_4 intensity (*n* = 80, *r* = 0.24, *p* = 0.03) and T2 intensity (*n* = 80, *r* = 0.31, *p* < 0.01); (3) O1T2_8 lateralization score positively correlated with O1T2_8 intensity (*n* = 80, *r* = 0.24, *p* = 0.03). Additionally, identification score was positively correlated with O2T2_4 lateralization score (*n* = 80, *r* = 0.24, *p* = 0.03) but negatively correlated with O1T1_8 lateralization score (*n* = 81, *r*=-0.24, *p* = 0.03). The importance of olfaction positively correlated with O2T1_4 lateralization score (*n* = 80, *r* = 0.23, *p* = 0.02).

## Discussions

The present study examined whether lateralization performance significantly improved when “olfactory” odors were mixed with small amount of irritating (trigeminal) compounds. The results consistently showed no significant improvement in lateralization performance with olfactory-trigeminal mixtures compared to performance with “olfactory” odors. Both “olfactory” odors and these mixtures resulted in significantly worse lateralization performance than “trigeminal” odors. It seems that a small degree of irritant was insufficient to significantly improve odor lateralization performance, challenging the “all or none” assumption. If this rule held true, lateralization performance would have significantly improved even with a very low level of trigeminal irritation added to an “olfactory” odor. However, our results did not support this. Instead, our findings suggested that odor lateralization in response to olfactory-trigeminal mixtures is more likely to follow an “accumulative pattern”, where only sufficient activation of the trigeminal system significantly enhances odor lateralization performance. Consequently, if a group of healthy participants consistently shows better lateralization performance with an unknown odor (i.e., significantly higher scores or a higher percentage reaching the above-chance threshold compared to a selectively “olfactory” odor), it likely contains substantial irritating compounds. However, if the odorant does not show this improvement in the same group, it does not necessarily mean the odor is completely devoid of trigeminal irritation. Taken together, the lateralization task lacks its sensitivity in detecting odors with a low degree of trigeminal compounds among more selective olfactory odors and appears to be a conservative measure of trigeminal activation. However, the task can still serve as a screening tool for identifying odors with substantial irritating compounds by comparing group-level performance with that of a selective olfactory odor.

It is important to acknowledge that commercial products were used in this study, as it was part of a larger project focused on examining everyday fragrances and their impact on various behavioral outcomes, such as memory. As a result, the “olfactory” and “trigeminal” odors used were not completely pure. For example, O1 was categorized as a relatively “olfactory” odor because participants did not perceive it as irritating. However, O1 contained small amounts of a lavender odorant with various components like camphor (associated with a menthol smell), which could activate the trigeminal nerve to some degree [18]. However, even when mixed with 4% and 8% of T1 or T2, resulting in greater trigeminal activation, lateralization performance still did not significantly improve and remained statistically worse than with T1 and T2 alone. While the odors may not have been ideal, this result still suggests that a small amount of irritating (trigeminal) compounds was insufficient to significantly enhance lateralization performance, indicating that the lateralization task is insensitive to low trigeminal activation. Although this pattern was replicable across four sets of odor combinations (O1 and T1, O1 and T2, O2 and T1, O2 and T2), future studies using more ideal odors (e.g., PEA for olfactory odor and CO2 for trigeminal odor) are needed to confirm these results. Further, the presence of trigeminal components in the relatively “olfactory” odors may explain why lateralization performance was better with O1 than with O2. Again, this is likely because O1 contains a lavender odorant with various components that may activate the trigeminal nerve slightly more than O2, which has passion fruit and vanilla scents, with vanilla being considered a more selectively “pure” odorant in previous studies [1, 4].

Regarding the variation of trigeminal sensitivity within the study sample, approximately 25% of participants failed to lateralize T1 or T2 significantly beyond chance (≥ 15), while around 30% succeeded in lateralizing O1 or O2 significantly above chance. Similar variation has been reported in previous studies [7]. For example, a study screened 152 healthy participants and found that 19 could lateralize the selectively olfactory odor PEA significantly above chance [7]. Importantly, even with such variations of trigeminal sensitivity, the performance of this subgroup did not contradict the main findings: their lateralization performance of 4% and 8% trigeminal irritants was comparable to “olfactory” odors (0% irritants) but significantly worse than “trigeminal” odors (100% irritants). There was one exception: for participants unable to lateralize trigeminal odors beyond chance, post hoc tests indicated that lateralization performance with 100% irritants was not significantly higher than with 0% trigeminal irritants, though with a p-value (0.051) approaching significance. Considering that most results from this subset align with the overall sample, and the only non-significant post hoc result may relate to the small subset sample size of 31, it is reasonable to conclude that the odor lateralization task is conservative and lacks sensitivity in identifying odors with small degrees of trigeminal compounds among more selective olfactory odors. Furthermore, the observed individual differences raise questions about whether the odor lateralization paradigm accurately measures trigeminal sensitivity. Exploring alternative models, such as electrophysiological techniques, could provide further insights [19].

We found positive correlations between O1T1 and O1T2 intensity and their respective lateralization scores, consistent with prior research linking odor intensity and localization performance [12, 20]. Although the underlying mechanism is unknown, intensity ratings partly reflect the sensitivity at the mucosal level in the nasal cavity, and thus influence the degree of activation in the trigeminal system. Notably, O1T1 and O1T2 lateralization performances correlated with mixed or “trigeminal” odor intensity but not “olfactory” odor intensity, highlighting the stronger link between lateralization ability and trigeminal rather than olfactory sensations. This is similar to the study by Croy et al. [7], which revealed that participants who were able to lateralize PEA significant above chance had enhanced activation in trigeminal areas but not olfactory areas compared to controls. When it comes to the relationship between lateralization performance and olfactory identification, the results were heterogeneous. Olfactory identification scores positively correlated with O2T2_4 lateralization performance but negatively correlated with O1T1_8 lateralization performance. These inconsistencies might be associated with the relatively small variation among healthy participants, making it difficult to detect a consistent correlation. More data are needed in future studies. Regarding the importance of olfaction, it only positively correlated with the O2T1_4 lateralization score, but did not correlate with the scores from the other 11 odors. This is similar to previous research, which also found no significant correlation between lateralization performance and the importance of olfaction [21]. Unlike olfactory function, individuals who value their sense of smell may not necessarily experience significant benefits in terms of trigeminal sensation [22].

Some limitations should be discussed in our study. Firstly, the odors used were not “pure” olfactory or trigeminal odors, as discussed earlier. Future studies should use more ideal odors (e.g., PEA for olfactory, CO2 for trigeminal) to test the replicability of the present results. Secondly, our study focused on the question whether addition of a small degree of trigeminal compound to olfactory odors would significantly enhance lateralization performance. Future studies with a concentration gradient would allow for a better understanding of the concentration-response relationship. Lastly, to ensure smoother data collection and minimize participant burden, we conducted tests in three sessions over two weeks. We anticipated olfactory odors as the most challenging for lateralization, so we started with trigeminal odors or trigeminal-olfactory mixtures to boost participant confidence and motivation. As a result, trigeminal compounds were tested in the first two sessions, with olfactory odors in the third session. This resulted in a dropout during the third session, with only 53 participants completing the lateralization task for O1 and O2. Although there were no significant demographic differences between dropout and non-dropout groups, and we accounted for missing data using the GLMM with Satterthwaite method, future studies would benefit from a larger sample size.

## Conclusions

lateralization performance for odors with small amounts of trigeminal compounds was comparable to selective “olfactory” odors but significantly worse than “trigeminal” odors. Trigeminal lateralization is more likely to follow an “accumulative” pattern rather than an “all or none” rule. The presently used lateralization task with 20 trials may lack sensitivity in identifying odors with low degrees of trigeminal irritation among more selective olfactory odors, yet without excluding the possibility of trigeminal system activation. However, the task can still serve as a screening tool for identifying trigeminal odors with substantial irritating compounds by comparing group-level performance with that of a selective olfactory odor. Furthermore, odor intensity plays an important role in odor lateralization.
